# Iron bioavailability and cardiopulmonary function during ascent to very high altitude

**DOI:** 10.1183/13993003.02285-2019

**Published:** 2020-09-17

**Authors:** David A. Holdsworth, Matthew C. Frise, Josh Bakker-Dyos, Christopher Boos, Keith L. Dorrington, David Woods, Adrian Mellor, Peter A. Robbins

**Affiliations:** 1 Dept of Physiology, Anatomy and Genetics, University of Oxford, Oxford, UK; 2 Royal Centre for Defence Medicine, Queen Elizabeth Hospital, Birmingham, UK; 3 Institute for Sport, Physical Activity and Leisure, Leeds Beckett University, Leeds, UK; 4 Dept of Postgraduate Medical Education, Bournemouth University, Bournemouth, UK

## Abstract

More than one hundred million people reside worldwide at altitudes in excess of 2500 m above sea level. In the millions more who sojourn at high altitude for recreational, occupational or military pursuits, hypobaric hypoxia drives physiological changes affecting the pulmonary circulation, haematocrit and right ventricle (RV) [1]. Coincident with these, maximal left ventricular (LV) stroke volume (SV) falls [2], with a reduction of 20% reported after a 2-week stay at 4300 m [3]. A rise in heart rate (HR) compensates at rest and during submaximal exercise but is insufficient during maximal intensity exercise, constraining maximal cardiac output (CO). Previously, it was considered that a reduction in plasma volume or a direct effect of hypoxia on LV myocardial contractility were probably responsible [4]. More recently it has been suggested that increased RV afterload may be of greater importance [5].

## Abstract 2

**Intravenous iron supplementation at sea level is associated with enhanced stroke volume and higher *S*_pO_2__ on ascent to very high altitude (5100 m). These effects appear to result from reduced pulmonary vascular resistance and improved right heart function.** https://bit.ly/2VQX5fR

*To the Editor*:

More than one hundred million people reside worldwide at altitudes in excess of 2500 m above sea level. In the millions more who sojourn at high altitude for recreational, occupational or military pursuits, hypobaric hypoxia drives physiological changes affecting the pulmonary circulation, haematocrit and right ventricle (RV) [1]. Coincident with these, maximal left ventricular (LV) stroke volume (SV) falls [2], with a reduction of 20% reported after a 2-week stay at 4300 m [3]. A rise in heart rate (HR) compensates at rest and during submaximal exercise but is insufficient during maximal intensity exercise, constraining maximal cardiac output (CO). Previously, it was considered that a reduction in plasma volume or a direct effect of hypoxia on LV myocardial contractility were probably responsible [4]. More recently it has been suggested that increased RV afterload may be of greater importance [5].

Hypoxic pulmonary vasoconstriction (HPV) contributes significantly to increased RV work and pulmonary hypertension during alveolar hypoxia [6]. In healthy iron-replete individuals, intravenous (*i.v*.) iron attenuates HPV [7, 8], tending to reduce RV afterload. We hypothesised that *i.v.* iron would improve cardiopulmonary function during ascent to very high altitude through this action upon the pulmonary vasculature, with or without a direct effect on the heart.

We conducted a randomised, controlled, double-blind, clinical physiology study. 18 British Armed Forces personnel (17 male, 1 female) volunteered; one was excluded because of abnormal baseline iron indices. Participants were randomised to receive either 1 g ferric carboxymaltose (Ferinject), or saline control, as a single infusion. 2 weeks later, participants flew to Kathmandu, Nepal, at an altitude of 1400 m, were driven to 2600 m (day 4), trekked to 3800 m (day 5), 4100 m (day 7), and then 5100 m (day 10). Serial measurements of iron indices, peripheral oxyhaemoglobin saturation (*S*_pO_2__), and transthoracic echocardiographic parameters (VividQ, GE, Boston, MA, USA) were recorded at rest.

Stroke volume was estimated by multiplying LV outflow tract (LVOT) velocity-time integral (VTI) by LVOT cross-sectional area, and CO by multiplying SV and HR. Both were then normalised to body surface area in m^2^ (BSA; Mosteller formula).

Right ventricular systolic pressure (RVSP) was estimated from the peak velocity of the tricuspid regurgitation jet [1, 5, 7–9]. The LV and RV indices of myocardial performance (LIMP and RIMP) and tricuspid annular planar systolic excursion (TAPSE) were measured. Pulmonary vascular resistance (PVR) was estimated using the Abbas method [9]. Between-group differences in responses were analysed using mixed-effects modelling (SPSS Statistics version 25, IBM). Ethical approval was given by the Ministry of Defence Research Ethics Committee, all participants provided written informed consent, and the study was registered with ClinicalTrials.gov (NCT03707249).

The groups were well matched at baseline. Comparisons for the iron group *versus* controls were as follows: mean±sd age 35.5±8.2 *versus* 36.1±7.7 years; body mass index 24.8±1.0 *versus* 24.6±2.0 kg·m^−2^; and BSA 2.02±0.06 *versus* 1.99±0.04 m^2^. No adverse infusion-related events occurred. One participant in the control group did not ascend beyond 4100 m due to severe gastrointestinal symptoms; all available data for this participant were included in the analysis.

Changes in iron indices, haematological parameters and cardiopulmonary variables are illustrated in figure 1. Ferritin and hepcidin were elevated in the iron group, with a corresponding reduction in the rise in both erythropoietin and soluble transferrin receptor (sTfR).

**FIGURE 1:**
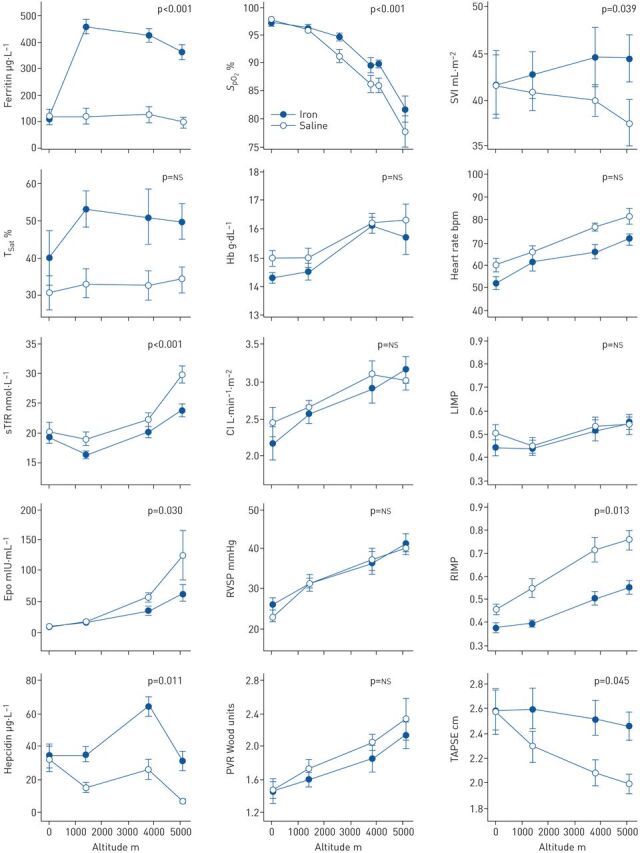
Variation with altitude of iron indices, haematological parameters and cardiopulmonary physiological variables. Sea-level data were acquired immediately prior to infusion of iron or saline. Data are plotted as mean±sem. The p-values given are for the interaction between group and altitude, that is, whether iron administration altered the change from sea level to maximum altitude. *S*_pO_2__: peripheral oxyhaemoglobin saturation; SVI: stroke volume index; T_Sat_: transferrin saturation; Hb: haemoglobin concentration; sTfR: soluble transferrin receptor; CI: cardiac index; RIMP and LIMP: RV and LV indices of myocardial performance (combined measures of the efficiency of ventricular filling and ejection; higher values indicate more significant impairment); Epo: erythropoietin; RVSP: right ventricular systolic pressure; PVR: pulmonary vascular resistance; TAPSE: tricuspid annular planar systolic excursion.

The prior administration of iron significantly attenuated the progressive fall in *S*_pO_2__ seen with increasing altitude (absolute difference in desaturation 5.5%, 95% CI 2.5–8.4%; p<0.001). Iron also abolished the normal fall in SV observed with increasing altitude. The mean between-group difference in the change in stroke volume index (SVI) was 6.2 mL·m^−2^ (95% CI 0.31–12.2 mL·m^−2^; p=0.039).

In the control group, LIMP, RIMP and TAPSE all worsened significantly with increasing altitude. LIMP rose by 0.08 (95% CI 0.003–0.16; p=0.043), RIMP rose by 0.31 (95% CI 0.24–0.38; p<0.001), and TAPSE fell by 0.55 cm (95% CI 0.27–0.83 cm; p<0.001). When comparing the iron group with controls, the degree of impairment in RIMP and TAPSE was reduced by 0.14 (95% CI 0.03–0.24; p=0.013) and 0.41 cm (95% CI 0.01–0.82 cm; p=0.045), respectively. However, the iron group showed no difference in the deterioration in LIMP (95% CI for between group difference −0.07–0.16; p=0.41) nor the rise in RVSP on ascent (95% CI −7.6–4.0 mmHg; p=0.51).

Interestingly, we found that iron supplementation was associated with augmented SV in the absence of a difference in RVSP. Had PVR remained similar in both groups, the higher SV of the iron group would be expected to have associated with a higher RVSP. In fact, RVSP responses were similar and there appeared to be a trend towards a lower PVR in the iron group, although this was not statistically significant (95% CI −0.58–0.23 Wood units; p=0.38). However, a strong negative correlation was evident between the change in SVI and the change in PVR (Pearson's *r*=−0.72; p=0.003), implying a close relationship between increased RV afterload and falling SV.

Reduced PVR might be a direct result of increased iron bioavailability, as previously described [7, 8], or may result from improved oxyhaemoglobin saturation. The latter would also act to reduce HPV as the result of a corresponding increase in mixed venous oxygen tension. The latter is a significant stimulus for HPV, albeit less so than alveolar oxygen tension [10]. Both mechanisms are biologically plausible, as is the putative mechanism for increased oxygenation in the iron group: that an iron-mediated reduction in HPV promotes ventilation/perfusion matching. The finding that RV, but not LV, function was enhanced in the group given iron also seems likely to reflect reduced RV work secondary to attenuated HPV and consequently reduced PVR.

An alternative explanation would be that iron acted to augment the ventilatory response to hypobaric hypoxia. We were not able to measure ventilation as part of the expedition. Whilst there is good reason to believe iron bioavailability might affect pulmonary ventilation *via* an action on the hypoxia inducible factor pathway within carotid body glomus cells [11], no human study has detected such a phenomenon [8, 12]. Moreover, the expected direction of effect is for iron to diminish alveolar ventilation rather than augment it.

The links between iron, erythropoiesis and oxygen homeostasis are complex [13]. Erythropoietin is under transcriptional regulation by both hypoxia and iron [12], so the attenuated erythropoietin rise in the iron group will reflect some combination of both a direct action of iron and improved renal oxygenation. The rise in sTfR, levels of which reflect the balance between iron supply and erythropoietic activity [14], was similarly attenuated in the iron group, reflecting some combination of greater iron bioavailability and lower stimulation of the bone marrow by erythropoietin. Both iron and hypoxia regulate expression of hepcidin, the key hormone regulating iron homeostasis; the effect of hypoxia is indirect, mediated downstream of marrow stimulation [13]. The effect of prior iron infusion on iron bioavailability in the present study was so marked that it lifted the heavy suppression of hepcidin seen at 5100 m in the control group.

A role for *i.v.* iron therapy is well established in chronic heart failure [15]. Our findings support the view that manipulation of iron bioavailability should be explored more broadly in conditions that feature increased PVR, ventilation/perfusion mismatch, or right heart dysfunction, including right heart failure, acute pulmonary embolism, high altitude pulmonary oedema, adult congenital heart disease, chronic thromboembolic pulmonary hypertension and COPD.

## Shareable PDF

10.1183/13993003.02285-2019.Shareable1 This one-page PDF can be shared freely online.
Shareable PDF ERJ-02285-2019.Shareable
