# Supplementary material for: Iron bioavailability and cardiopulmonary function during ascent to very high altitude
Source: Eur Respir J. 2020 Sep 17;56(3):1902285. doi: 10.1183/13993003.02285-2019 (PMC7494841; doi:10.1183/13993003.02285-2019)
Supplement: Supplementary file 1 [file ERJ-02285-2019.Shareable.pdf]

AGORA
RESEARCH LETTER

SHAREABLE PDF

Iron bioavailability and cardiopulmonary function during ascent to very high altitude

David A. Holdsworth[1,2], Matthew C. Frise [1], Josh Bakker-Dyos[2], Christopher Boos[3,4], Keith L. Dorrington[1], David Woods[2,3], Adrian Mellor[2,3] and Peter A. Robbins [1]

Affiliations: [1]Dept of Physiology, Anatomy and Genetics, University of Oxford, Oxford, UK. [2]Royal Centre for Defence Medicine, Queen Elizabeth Hospital, Birmingham, UK. [3]Institute for Sport, Physical Activity and Leisure, Leeds Beckett University, Leeds, UK. [4]Dept of Postgraduate Medical Education, Bournemouth University, Bournemouth, UK.

Correspondence: David A. Holdsworth, University of Oxford, Dept of Physiology, Anatomy and Genetics, Sherrington Building, Parks Road, Oxford OX1 3PT, UK. E-mail: david.holdsworth@dpag.ox.ac.uk

@ERSpublications
Intravenous iron supplementation at sea level is associated with enhanced stroke volume and higher S_{pO_2} on ascent to very high altitude (5100 m). These effects appear to result from reduced pulmonary vascular resistance and improved right heart function. https://bit.ly/2VQX5fR

Cite this article as: Holdsworth DA, Frise MC, Bakker-Dyos J, *et al.* Iron bioavailability and cardiopulmonary function during ascent to very high altitude. *Eur Respir J* 2020; 56: 1902285 [https://doi.org/10.1183/13993003.02285-2019].

This single-page version can be shared freely online.

To the Editor:

More than one hundred million people reside worldwide at altitudes in excess of 2500 m above sea level. In the millions more who sojourn at high altitude for recreational, occupational or military pursuits, hypobaric hypoxia drives physiological changes affecting the pulmonary circulation, haematocrit and right ventricle (RV) [1]. Coincident with these, maximal left ventricular (LV) stroke volume (SV) falls [2], with a reduction of 20% reported after a 2-week stay at 4300 m [3]. A rise in heart rate (HR) compensates at rest and during submaximal exercise but is insufficient during maximal intensity exercise, constraining maximal cardiac output (CO). Previously, it was considered that a reduction in plasma volume or a direct effect of hypoxia on LV myocardial contractility were probably responsible [4]. More recently it has been suggested that increased RV afterload may be of greater importance [5].

Copyright ©ERS 2020.. This version is distributed under the terms of the Creative Commons Attribution Licence 4.0.
